# Reference for Normal Diameters of the Abdominal Aorta and Common Iliac Arteries in the Saudi Population

**DOI:** 10.7759/cureus.30695

**Published:** 2022-10-26

**Authors:** Mohammad Wazzan, Ahmed Abduljabbar, Amr Ajlan, Rani Ahmad, Turki Alhazmi, Ayman Eskandar, Khalid Khashoggi, Farah Alasadi, Shahad Howladar, Yousra Alshareef

**Affiliations:** 1 Radiology, King Abdulaziz University Faculty of Medicine, Jeddah, SAU; 2 Medical Imaging, Umm Alqura University, Makkah, SAU; 3 Medical Imaging, Umm Al-Qura University, Makkah, SAU; 4 Radiology, King Abdulaziz Medical City, Jeddah, SAU; 5 Radiology, King Faisal Specialist Hospital and Research Center, Jeddah, SAU; 6 Radiology, King Abdulaziz University Hospital, Jeddah, SAU

**Keywords:** iliac artery, ct scan, aorta, aaa, abdominal aortic aneurysm

## Abstract

The abdominal aorta is the largest artery in the abdomen. It then bifurcates giving the two common iliac arteries. Knowing the normal abdominal aorta diameter is a basis for diagnosing abdominal aortic aneurysms (AAAs) and subsequently developing an optimal management plan. In order to diagnose AAA, one must have a reference for the normal abdominal aortic diameter that represents the anatomical variation in the population being studied. The aim of this research is to establish normal abdominal aortic diameters in the Saudi population.

## Introduction

The abdominal aorta is the largest artery in the abdomen; it starts from the diaphragm as a continuation of the descending aorta and gives off branches to supply multiple organs. It then bifurcates into the two common iliac arteries. The abdominal aorta length is approximately 13 cm [1]. Knowing the normal abdominal aorta diameter is a basis for diagnosing AAAs and subsequently developing an optimal management plan.

An AAA is defined as a dilatation in the abdominal aorta of 3 cm or greater [2]. However, there is no specific definition of AAA that has been widely agreed upon, it depends on the abdominal aortic diameter which is affected by age, sex, and body mass [3]. In order to diagnose AAA, one must have a reference for the normal abdominal aortic diameter that represents the anatomical variation in the population being studied. References used in Saudi medical practice are based on data from western literature. No previous studies of normal abdominal aortic diameter were done in Saudi Arabia. The aim of this research is to establish a normal abdominal aortic diameter reference range in the Saudi population. 

## Materials and methods

We examined contrast-enhanced CT scan findings of 2,100 patients in a tertiary hospital in Saudi Arabia, retrospectively studied. A sample size of 399 subjects met the inclusion criteria. All subjects were low-risk adults (18 years old and above) of both genders. Any patients with atherosclerotic changes, neoplastic disease, inflammatory disease, or infectious disease were excluded from the study. The diameters of the suprarenal abdominal aorta (3 cm above renal artery branch), infrarenal aorta (3 cm below renal artery branch), and right and left common iliac artery were measured in three different views (coronal, sagittal, and transverse reformats).

CT imaging protocol

The CT studies were performed on one of three CT scanners ranging from 32-slice to 128-slice multidetector computed tomography (MDCT) Siemens scanner. The protocol was 3-mm slice thickness, 140 kVp, 250-ms exposure time, and a 350-mm field of view. The CT images were acquired in the craniocaudal direction in a single breath-hold for all examinations. For interpretation, the images were transferred to the PACS workstation.

Statistical analysis

The aortic diameter was calculated as the sum of the diameter of the sagittal plane, transverse plane, and anteroposterior plane divided by three. All statistical analysis was done using SPSS version 23.0 software (IBM SPSS, Chicago, IL). Student’s t-test was used to evaluate the difference in diameter between males and females. One-way ANOVA was used to evaluate the difference in diameter with age. A p-value of < 0.05 was considered statistically significant.

## Results

Three hundred ninety-nine people were enrolled in our screening project. The data were gathered from 160 males and 239 females, with a mean age of 50.8 years (range 18 to 98).

We analyzed the normal diameter of the abdominal aorta and the bilateral iliac arteries. The mean diameter at the level of the suprarenal aorta was 1.68 cm and 1.36 cm at the level of infrarenal aorta. The diameter of the right iliac artery was 0.90 cm and the diameter of the left iliac artery was 0.88 cm. There were significantly larger diameters in the male population compared with the female population on Student’s t-test except for the left and right common iliac arteries (Table 1).

**Table 1:** Normal diameter of abdominal aorta (cm) at different anatomical level (N=399). SD, standard deviation. *With Student’s t-test.

Anatomic Level	Male (n=160) Mean±SD	Female (n=239) Mean±SD	P-value*	Total Mean±SD
Supra-renal aorta	1.75±0.27	1.63±0.32	.000	1.68±0.31
Infra-renal aorta	1.43±0.17	1.32±0.23	.000	1.36±0.21
Right common iliac	0.93±0.16	0.88±0.51	0.214	0.90±0.41
Left common iliac	0.92±0.16	0.86±0.47	0.131	0.88±0.38

We also analyzed the normal diameter of each level with the increase in age. Only the diameters of the suprarenal and infrarenal arteries increased with age (Table 2).

**Table 2:** Normal diameter of abdominal aorta (cm) with age (N=399) (mean±standard deviation) Statistical analysis with ANOVA (p-value < 0.05).

Age	N	Supra-renal aorta	Infra-renal aorta	Right common iliac	Left common iliac
29 ≤	48	1.38±0.22	1.27±0.42	0.93±1.0	0.92±0.96
30-39	61	1.52±0.23	1.50±01.7	0.87±0.43	0.87±0.38
40-49	68	1.63±0.22	1.36±0.13	0.86±0.14	0.84±0.13
50-59	92	1.74±0.20	1.39±0.17	0.87±0.12	0.86±0.13
60 ≥	130	1.82±.26	1.42±.15	.93±.19	.91±.17

## Discussion

Many factors increase the risk of AAA including age, sex, family history, hypertension, hypercholesterolemia, cerebrovascular disease, coronary artery disease, and smoking. A study showed that a larger abdominal aortic diameter also comprises a higher risk of developing AAA in the future [4]. Knowing the normal abdominal aortic diameter is essential to diagnosing aortic pathologies such as AAA and it is especially crucial for deciding when to surgically intervene. AAA is diagnosed when the abdominal aortic diameter is 3.0 cm or greater or if its diameter is 50% or two standard deviations greater than its mean diameter [5-7].

In this study, we provide a reference for normal abdominal aortic diameter stratified by age and sex in a low-risk population in Jeddah, Saudi Arabia. There are different modalities to measure the aortic diameter which include computed tomography, ultrasound, and magnetic resonance imaging. There is no gold standard imaging modality to measure the aortic diameter and to diagnose AAA, however, The CT scan is more accurate in measurements and is essential prior to any intervention, while ultrasound is cost-effective and easily accessible but has its limitations in that it is operator-dependent, and images can be of low quality due to different factors related to patients such as bowel gas or obesity [8-10]. A previous study showed that ultrasound measurements were smaller than CT measurements by an average of 0.27 cm [11]. However, another study done in Sweden showed that the US gave larger measurements of abdominal aortic diameter compared to CT [10]. This could be related to technique differences. In this study computed tomography was used as the modality of choice.

The CT scans studied in this research were performed for different clinical indications, none of the subjects were exposed to excess radiation solely for this research. All subjects enrolled in this study were adults, with no comorbid conditions such as diabetes mellites, hypertension, or cardiovascular diseases and they were all anonymously chosen.

No studies were found assessing the normal diameter of the abdominal aorta in Jeddah, Saudi Arabia. However, several studies were done outside Saudi Arabia, some of which used computed tomography as their modality of measurement while others used ultrasonography.

Previous studies have shown an increase in abdominal aortic diameter with increased age and body surface area (BSA), and larger abdominal aortic diameter in men compared to women [12-14]. In our study, age and sex were the only variables included. For example, a study done in Massachusetts in 2013 showed larger abdominal aortic diameter in men compared to women; the average diameter was 19.3 mm and 18.7 mm in men, and 16.7 mm and 16.0 mm in women for infrarenal abdominal aorta and lower abdominal aorta, respectively [13]. Another study done in India showed a similar correlation [15]. In the current study, the diameters of the suprarenal and infrarenal aorta were significantly greater in the male population compared to the female population as seen in the previous studies; with a mean diameter of the suprarenal aorta of 1.75±0.27 cm in men and 1.63±0.32 cm in women and a mean diameter of the infrarenal aorta of 1.43±0.17 cm in men compared to 1.32±0.23 cm in women.

Age was also found to have a positive correlation with the suprarenal and infrarenal abdominal aortic diameters (p-value <0.05), as demonstrated in previous studies. A study done in Korea measured the abdominal aorta at different levels and both common iliac arteries using ultrasound. It showed significantly larger diameters in men compared to women except for the left iliac artery. All diameters were increased with age [8]. The right and left common iliac arteries in our current study, however, showed no significant change with age or sex. The mean diameter of the right common iliac is 0.93±0.16 cm in males and 0.88±0.51 cm in females, and the mean diameter of the left common iliac artery is 0.92±0.16 cm in males and 0.86±0.47 cm in females.

## Conclusions

The mean diameter at the level of the suprarenal aorta was 1.68 cm and 1.36 cm at the level of the infrarenal aorta. The diameter of the right iliac artery was 0.90 cm and the diameter of the left iliac artery was 0.88 cm. There were significantly larger diameters in the male population compared with the female population. Only the diameters of the suprarenal and infrarenal arteries increased with age. The suprarenal and infrarenal abdominal aortic diameters were found to be significantly larger in men than women and showed a significant increase with age. The right and left common iliac arteries showed no significant correlation with age or sex.
